# Approaches for the Treatment and Resource Utilization of Electroplating Sludge

**DOI:** 10.3390/ma17071707

**Published:** 2024-04-08

**Authors:** Song Guo, Huimin Wang, Xiaoming Liu, Zengqi Zhang, Yu Liu

**Affiliations:** 1 School of Metallurgical and Ecological Engineering, University of Science and Technology Beijing, Beijing 100083, China; 18513261866@163.com (S.G.); m202210235@xs.ustb.edu.cn (H.W.); 2 State Key Laboratory of Advanced Metallurgy, School of Metallurgical and Ecological Engineering, University of Science and Technology Beijing, Beijing 100083, China; 3 China International Engineering Consulting Corporation, Beijing 100048, China; itvrly@163.com

**Keywords:** electroplated sludge, hazardous solid waste, resource utilization, disposal process, pollutant control, technology comparison, waste utilization

## Abstract

The disposal of electroplating sludge (ES) is a major challenge for the sustainable development of the electroplating industry. ESs have a significant environmental impact, occupying valuable land resources and incurring high treatment costs, which increases operational expenses for companies. Additionally, the high concentration of hazardous substances in ES poses a serious threat to both the environment and human health. Despite extensive scholarly research on the harmless treatment and resource utilization of ES, current technology and processes are still unable to fully harness its potential. This results in inefficient resource utilization and potential environmental hazards. This article analyzes the physicochemical properties of ES, discusses its ecological hazards, summarizes research progress in its treatment, and elaborates on methods such as solidification/stabilization, heat treatment, wet metallurgy, pyrometallurgy, biotechnology, and material utilization. It provides a comparative summary of different treatment processes while also discussing the challenges and future development directions for technologies aimed at effectively utilizing ES resources. The objective of this text is to provide useful information on how to address the issue of ES treatment and promote sustainable development in the electroplating industry.

## 1. Introduction

**Figure 1:**
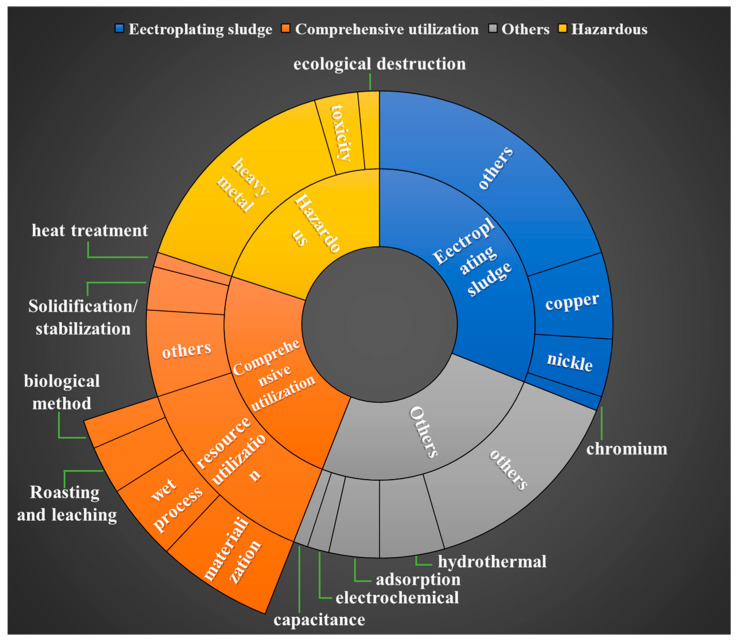
Research hotspots and framework for the comprehensive utilization of ESs.

Electroplating technology is widely applied in various fields, such as mechanical manufacturing, electronic instruments, and metal materials, thereby facilitating the rapid growth of the economy [1]. However, electroplating processes generate a significant amount of wastewater, which is transformed into ES through acid–base neutralization, coagulation, and sedimentation. According to statistics, there are approximately 200,000 electroplating enterprises in China, generating an annual output of 5 billion cubic meters of electroplating wastewater and discharging over 13 million tons of electroplating sludge [2]. The annual discharge of electroplating sludge in the EU is approximately 100,000 tons, whereas the total discharge of sludge in other countries and regions worldwide exceeds 1 million tons [3]. Consequently, the electroplating industry is widely recognized as one of the most polluting industries worldwide [4]. During electroplating processes, various organic substances, including surfactants and complexing agents (e.g., citric acid and ethylenediaminetetraacetic acid), are commonly added [5]. While these additions enhance product quality in electroplated products, they also increase challenges associated with treating electroplating wastewater and sludge while complicating ES characteristics. ESs typically exhibit a moisture content ranging from 75% to 90% and a pH value between 7.5 and 8.0, along with an ash content exceeding 76%. Additionally, ESs have a complex composition, comprising various heavy metals (such as Cu, Zn, Ni, Cr, and Pb), calcium salts, organic compounds, and other toxic/harmful substances [6,7,8]. As a consequence, it is categorized as hazardous solid waste. Improper disposal and indiscriminate dumping of ES not only occupy a significant amount of land resources but also pose a severe threat to the ecological environment and human health. Heavy metals in the sludge can migrate due to weathering and rainwater leaching, leading to environmental contamination through the sludge–soil–crop–human pathway and posing a substantial risk to human health [9,10]. As shown in Table 1, certain metals in ESs have higher grades than natural ores and are considered an economically viable renewable resource [11]. The global annual discharge of ES exceeds 10 million tons [12], and if not properly utilized, it would result in tremendous resource wastage. In modern society, where natural resources are gradually depleting, recovering resources from byproducts like ESs proves to be an effective approach for alleviating resource scarcity and promoting sustainable development within the electroplating industry [13,14]. Furthermore, the resource utilization of ESs aligns with global objectives toward carbon neutrality. In recent years, scholars have conducted comprehensive research on ESs, with key research areas illustrated in Figure 1.

The treatment of electroplating primarily involves processes such as solidification and stabilization, reduction, metal resource recovery, and high-value utilization of sludge materials. Given the hazardous nature and resource potential of ES, this paper provides a comprehensive review of the treatment and resource utilization of ES. It extensively outlines different utilization methods and progress, conducts comparative evaluations of various treatment technologies, and explores future developments in ES treatment techniques, offering new insights into the treatment and disposal of ES.

## 2. Methodology Adopted for Review

This study aims to comprehensively investigate the pretreatment, reduction, and resource utilization technologies of ES. It specifically focuses on the solidification technology for hazardous substances in ES, as well as hydrometallurgical and pyrometallurgical treatment processes, along with the application of ES in materialization. To achieve this objective, extensive and thorough literature searches were conducted across multiple authoritative databases such as Science Direct, Scopus, Web of Science, and Google Scholar. Due to its comprehensive coverage and usability within the field of ES research, Science Direct was utilized as the primary source for our search. Through keyword classification including “electroplating sludge”, “reduction”, “resource utilization”, “valuable element recovery”, “building materials”, “hydrometallurgy”, and” pyrometallurgy”, a systematic literature search was carried out. Using the keywords “electroplating sludge” and “resource utilization”, we initially searched for relevant English-language publications, resulting in approximately 1300 articles on ES. However, as our study specifically focuses on solidification, wet treatment, pyrometallurgical treatment, biological treatment, and material utilization of ES, many publications not directly related to these areas were included in the initial search results. Therefore, rigorous literature screening was conducted to exclude those that did not align with our research focus, which narrowed down the scope of the literature to approximately 400 articles. In subsequent literature screening processes, we meticulously scrutinized the abstracts of each publication to ensure their close relevance to our research topic and extracted key information from them. Following this round of screening, we acquired 46 samples on solidification treatment of ES, 68 samples on metallurgical processes, 30 samples on material utilization, and 35 samples on biological treatment. All selected publications have undergone peer review and possess high academic quality. We conducted comprehensive research and analysis based on these publications while exploring the reduction, harmlessness, and resource utilization technologies of ES. Additionally, we compared and analyzed the advantages and disadvantages of various treatment processes.

## 3. The Nature of Electroplating Sludge

### 3.1. Source, Composition, and Classification of Electroplating Sludge

The characteristics of ES vary significantly in different regions due to the diverse nature of wastewater generated by various electroplating processes, which necessitates distinct treatment methods. Acid–base neutralization and coagulation–precipitation methods are commonly employed for the treatment of electroplating wastewater [15,16]. In cases where the wastewater contains metals such as Cr and Ni, an excess alkaline solution is utilized to induce a reaction and form hydroxide precipitates, followed by solid–liquid separation. Zinc-containing electroplating wastewater undergoes precipitation at a pH value of around 8.5, resulting in the formation of Zn(OH)_2_; however, both excessively acidic or alkaline conditions can dissolve amphoteric Zn(OH)_2_ [17]. The precipitate obtained from treating electroplating wastewater using these techniques is referred to as ES [18]. Chemical methods are predominantly employed in industrial production for the treatment of ES, including coagulation and sedimentation and acid–base neutralization, as well as oxidation–reduction reactions.

Chinese electroplating enterprises demonstrate features including strong market demand, extensive geographic coverage, technological lagging, inadequate industry standards, and substantial environmental pollution. To enhance product quality, performance, and service life, electroplating is utilized. Chromium (Cr) coating offers high corrosion resistance, wear resistance, and visually appealing surface quality, making it widely applied in the electronic, automotive, and mechanical industries. However, its use in consumer products is limited due to the strong toxicity of Cr^3+^ and Cr^6+^. Zinc plating is a cost-effective method for protecting steel parts from atmospheric and marine corrosion. Nickel (Ni) provides good chemical stability in various environments, high hardness for easy polishing, and high light reflection properties ideal for decorative coatings. Tin (Sn) exhibits strong weldability and electrical conductivity, which are favored in electronic products requiring welding. The diverse range of coatings and treatment processes results in the presence of various heavy metals in ESs, including Au, Ag, Fe, Cu, Cr, Ni, and Zn, among others. The utilization of multiple complexing agents and surfactants in the electroplating industry adds to the complexity of sludge composition. Moreover, ES can also include considerable amounts of oil, lime, silica, cyanide, and other substances, along with residual elements like S, C, and Ca originating from wastewater [19,20]. Technological advancements and the utilization of reagents have significantly enhanced the quality of electroplating products, thereby leading to notable improvements in their overall performance. However, these advancements have also introduced novel challenges in the treatment of electroplating wastewater and sludge, consequently posing substantial risks to both the ecological environment and human health [21]. The types and compositions of common ES samples are presented in Table 2.

### 3.2. Hazards of Electroplating Sludge

**Figure 2:**
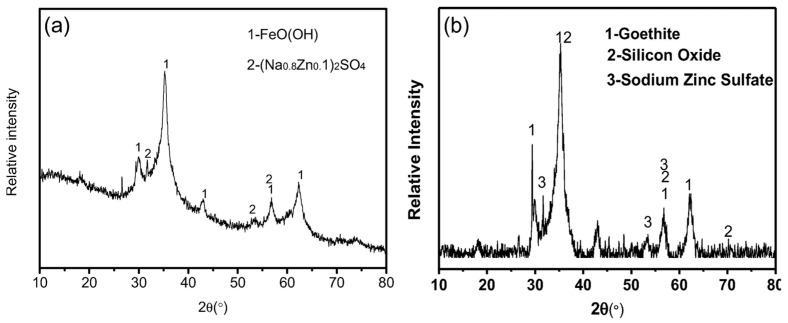
XRD image of (**a**) zinc-containing sludge and (**b**) mixed sludge [38,39]. (Elsevier copyright permission).

**Figure 3:**
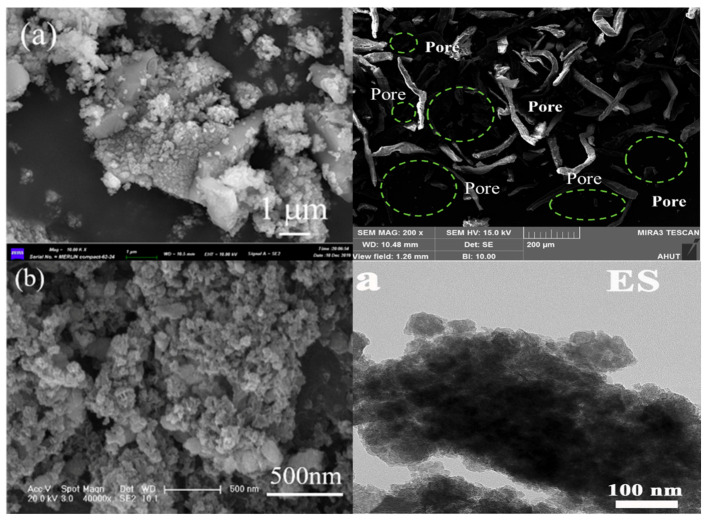
SEM images of different ES images [4,40,41,42]. (Elsevier and MDPI copyright permission).

ESs are characterized by a substantial discharge volume, high water content, complex composition, and unstable properties. Improper handling of this waste can result in significant ecological damage [34]. The composition of ES varies significantly depending on the specific processes involved. Currently, chemical precipitation is the most commonly employed method for treating electroplating wastewater, which results in heavy metals being present in an amorphous form within the generated sludge. Figure 2 and Figure 3 depict XRD and SEM images, respectively, showcasing some representative samples of ES. Inadequate disposal of this sludge containing toxic and harmful heavy metal ions can lead to severe environmental pollution, as rainwater or snow infiltrates into groundwater and causes soil microorganism mortality, soil compaction, and degradation [35], as well as crop and plant mortality that disrupts the ecological balance further. Weathering of ES surfaces can generate dust particles, which disperse heavy metals into the atmosphere, thereby causing air pollution. These pollutants may even pose a threat to human health and safety through their entry into the food chain [36]. Additionally, throughout the storage, transportation, and treatment stages of ES, inadequate management measures coupled with lax enforcement regulations have the potential to cause environmental pollution incidents. ESs are classified under hazardous wastes (HW17) containing toxic metallic elements according to China’s National List of Hazardous Wastes (2021 Edition) [37]. Stringent supervision regarding collection, storage, and disposal practices for these wastes is necessary to prevent secondary contamination.

The composition and nature of ESs exhibit significant variation, necessitating appropriate treatment measures. Currently, the focus of ES treatment methods primarily revolves around harm prevention and resource utilization [43]. Harm prevention encompasses stabilization and immobilization techniques, while resource utilization involves the recovery of valuable metals from ES or their transformation into high-value-added products. Numerous studies have yielded remarkable outcomes in the field of ES treatment. However, it is widely acknowledged that no existing method for treating ES can be deemed entirely safe, effective, or reliable. This article aims to explore several commonly employed methods for treating ES and provides insights into prospects regarding its resource utilization.

## 4. Electroplating Sludge Disposal Methods

### 4.1. Solidification/Stabilization Treatment

Solidification/stabilization is a critical method for treating ES and is widely employed for treating hazardous industrial solid waste. Solidification/stabilization technology refers to the addition of solidifying agents to ES, which results in the formation of a tightly bonded solid mass and the encapsulation of hazardous substances in sludge to prevent leaching. During the curing process of ESs, specific additives are incorporated based on the harmful substances in the sludge. Heavy metals and other toxic and hazardous substances are converted into compounds with reduced solubility, mobility, and toxicity, thus improving curing results. The solidified mass should exhibit excellent impermeability, resistance to leaching, resistance to dry mixing, resistance to freeze–thaw cycles, and adequate mechanical strength [44]. Commonly employed solidification/stabilization technologies include cement curing, lime curing, thermoplastic curing, melt curing, and self-cementing curing. The curing agents include cement, asphalt, glass, and water glass. This method has advantages such as low cost, high efficiency, easy implementation, simple operation, and wide applicability. However, finding an economical and effective solidifying agent remains a challenge. As the solidification time increases, the fluctuations in the surrounding environment increase the risk of heavy metals redissolving within the solidifying material, and the long-term stability of the solidification effect needs to be verified [38]. Additionally, solidification technology generates a significant amount of solid waste and secondary pollutants, while the valuable metals in sludge are not fully utilized, leading to the waste of resources.

In response to the issues in solidification and stabilization technology, researchers have conducted studies on the solidification mechanism and micromechanism of ES to seek more economically effective stabilizers, improve the stability of solidified products, and reduce the leaching of hazardous substances.

Chen et al. [45] utilized alkali-activated slag as a solidifying agent and conducted a systematic study on the effects of the liquid-to-solid ratio, modulus and dosage of water glass, and dosage of ES on the strength of the solidified body and the leaching rate of chromium ions. The solidified products were characterized, and it was determined that the appropriate dosage of ES in the alkali-activated slag binder is 15–18%. Additionally, the alkali-activated slag binder can convert Cr(VI) to Cr(III) without the need for a reducing agent. Orescanin et al. [46] cured ES at specific mass ratios of ES and calcium oxide and tested the stability and heavy metal content after leaching at different ratios. The findings indicate that an optimal ratio of ES to calcium oxide of 5:1 results in the highest stability of the solidified material, which is suitable for use in building materials. Yue et al. [47] successfully achieved the effective recovery of metals from sludge and the effective solidification of solidified products by adding fly ash during the melting and solidification of ES and SiO_2_. The experimental results indicated that the recovery rates of Zn, Pb, Cu, Cd, Cr, and Ni reached 33%, 96%, 33%, 79%, 81%, and 31%, respectively. Additionally, the leaching concentrations of Zn and Cu in the obtained quartz sand were 3.8 mg/L and 2.1 mg/L, respectively, while the leaching concentrations of the other metals were less than 1 mg/L. Asavapisit et al. [48] studied fly ash (Na_2_SiO_3_, Na_2_CO_3_) as a curing agent to solidify ES and examined the compressive strength and leaching characteristics of the solidified material. The results revealed that the inclusion of lime and two types of alkaline fly ash in ES can enhance the strength of cured cement. The optimum curing effect is achieved when the Na_2_SiO_3_ content is 4%, or the Na_2_CO_3_ content is 8%.

With the continuous progress and innovation of solid waste treatment technology, people’s awareness of environmental protection and resource recovery is constantly growing. Higher requirements have also been implemented for traditional solidification/stabilization technology. As new technologies continue to emerge, traditional solidification/stabilization technology is gradually being phased out and replaced.

### 4.2. Heat Treatment

Heat treatment involves the decomposition and oxidation of ES under high-temperature conditions, resulting in the elimination of water and volatile substances in the sludge, the destruction of toxic organic compounds, and a volume reduction in the treated ash residue to approximately 10% of the original sludge volume. This approach achieves both waste reduction and harmlessness. Common heat treatment processes include incineration, high-temperature melting, and example arcs.

Mao et al. [49] investigated the temperature dependence of Cr(VI) formation and reduction in the thermal treatment of chromium-containing sludge in the presence of CaO. The results indicated that CaO promotes the oxidation of Cr(III). In the presence of CaO, prolonging the heating time can suppress the oxidation of Cr(III) at low temperatures (200–400 °C). Additionally, at high temperatures (above 600 °C), the oxidation of Cr(III) can be reduced by elevated temperatures (1000–1200°C) or the addition of MgO. Using ion arc technology, Ramachandra et al. [50] examined the migration of heavy metals in ES under various atmospheric conditions and the subsequent transformation of ES to slag. The results indicated that a low oxygen content facilitated the migration of heavy metals. Chromium exhibited a high migration rate under nitrogen conditions, while nickel showed a high migration rate under hydrogen conditions. Wang et al. [51] investigated the use of thermal pretreatment on ES to enhance its bioleaching performance. The study demonstrated that thermal treatment significantly improved the bioleaching efficiency of sulfide ES. At a calcination temperature of 600 °C, the copper release efficiency dramatically increased from 59% to 100%, exhibiting the maximum promotion effect on bioleaching. However, high-temperature calcination at 800 °C strongly inhibited bioleaching, with a copper release rate of 46%.

Thermal treatment can minimize, stabilize, and prevent harm in ES to the greatest extent. However, due to the low calorific value and limited combustible components of ES, thermal treatment consumes a substantial amount of energy and requires high-temperature tolerance. Furthermore, the incineration of sludge can lead to secondary environmental pollution, thereby constraining the further application of thermal treatment technology.

## 5. Electroplating Sludge Resource Utilization

The ES resources contain abundant metal resources and have high economic value. Exploring the recovery and comprehensive utilization of metal elements in ESs is a research hotspot for the sustainable development of ecosystems. Consequently, the efficiency of extracting heavy metals using different processing methods was studied (Table 3). Common methods for the comprehensive utilization of ES resources include hydrometallurgical treatment, pyrometallurgical treatment, bioleaching, and material utilization, among others.

### 5.1. Hydrometallurgical Treatment

**Figure 4:**
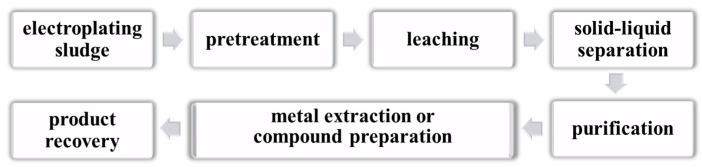
The general process of recovering valuable metals from ESs.

Hydrometallurgical technology involves dissolving valuable metals in sludge into leachate using leaching agents under optimal conditions. Subsequently, the leachate is purified and extracted to obtain metal products. This technology enables efficient recovery of metal resources with high efficiency in heavy metal recovery and stable processing effects. The hydrometallurgical recovery process of ESs primarily consists of two steps: the leaching of heavy metals and the recycling of leachate. Treatment methods for leachate include stepwise precipitation, solvent extraction, electrolytic deposition, ion exchange, and reduction. Hydrometallurgical technology comprises several steps, including pretreatment, leaching, solid–liquid separation, purification, and metal recovery (Figure 4). The main ES hydrometallurgical treatment methods include acid leaching and ammonia leaching.

#### 5.1.1. Acid Leaching Method

**Figure 5:**
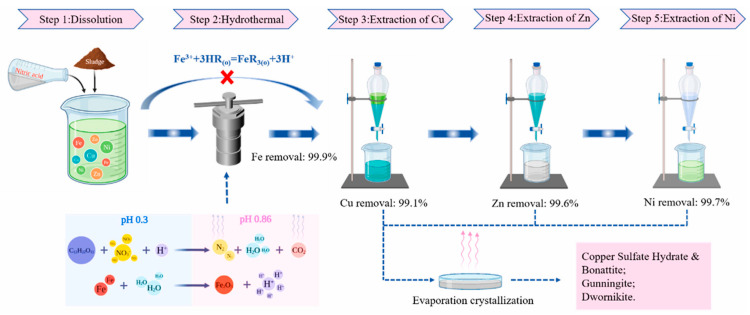
Acid leaching for the recovery of copper, zinc, and nickel from ES [63]. (Elsevier copyright permission).

The acid leaching method is extensively used in the hydrometallurgical treatment of ES. The majority of the precious metals in the sludge are predominantly present as hydroxides or oxides. Through acid leaching, the metallic components in the sludge can be dissolved into the solution, existing as soluble ions or complex ions, and subsequently separated and gathered. Currently, commonly utilized acidic leaching agents for ES leaching include sulfuric acid, nitric acid, hydrochloric acid, and aqua regia [53]. The leaching agent selection principle is based on thermodynamic feasibility, a high leaching rate, economic feasibility, and availability. The acid leaching process is illustrated in Figure 5.

The leaching effect of the acid leaching method on ES is influenced by various factors, including the type and concentration of acid, solid-liquid ratio, sludge type, leaching time, and temperature. Li et al. [64] utilized sulfuric acid as a leaching agent to investigate the impact of acid dosage on the leaching effect of Cu and Ni in sludge. The authors discovered that the optimal process conditions were achieved when 2 g of sludge with a particle size of d = 0.15 mm was mixed with 10 mL of 10% acid at room temperature and subjected to 0.5 h of oscillation. By acidifying 1 g of sludge with 5 mL of 10% sulfuric acid, the leaching rate of Cu and Ni in the ES exceeded 95%. Hoong Hooi Sim et al. [52] investigated the optimal concentration of sulfuric acid (H_2_SO_4_) for the removal of copper (Cu), nickel (Ni), and chromium (Cr) from the waste residue, as well as the residual organic matter after acid digestion. The optimal pH values for the precipitation of Cu and Ni were determined. The results indicated that the concentration of H_2_SO_4_ had a significant impact on the organic matter content of the sludge, with removal rates of 98.99%, 99.27%, and 97.22% for Cu, Ni, and Cr, respectively, when 30% H_2_SO_4_ was used. Lee et al. [65] studied the utilization of aqua regia as a leaching agent for treating ES containing tungsten carbide and cobalt. By examining the influence of temperature and reaction time on the leaching process, they found that this method achieved an almost 100% leaching rate for both cobalt and tungsten under suitable conditions. Li et al. [55] recovered Cu, Ni, Zn, Fe, and Cr from ES using a two-stage acid leaching process enhanced by ultrasonic waves, achieving recovery rates of 97.42%, 98.46%, 98.63%, 98.32%, and 100%, respectively. The metal elements mainly exist in the form of oxides and hydroxides in the ES, with the primary reaction occurring during the leaching process as follows:

MO_0.5n_ + nH^+^ → M^n+^ + 0.5nH_2_O (1)

M(OH)_n_+ nH^+^ → M^n+^ + nH_2_O (2)

nFe^3+^ + 3N(OH)_n_ → 3M^n+^ + nFe (OH)_n_ (3)

nCr^3+^ + 3N(OH)_n_ → 3M^n+^ + nCr (OH)_n_ (4)

where M represents Cu, Ni, Zn, Fe, and Cr, and N represents Cu, Ni, and Zn.

Numerous studies have demonstrated that the acid leaching method has the advantages of a short reaction time, high leaching efficiency, and cost-effectiveness. Nevertheless, acid leaching agents are often corrosive; therefore, corrosion resistance must be met. Moreover, the acid leaching process generates waste acid and wastewater laden with valuable metals, thereby posing a potential risk of secondary pollution. Furthermore, strong acid leaching lacks selectivity, as all metal oxides can theoretically undergo leaching within the leaching range defined by the redox potential of the system. Consequently, the composition of the leaching solution becomes intricate, rendering the separation and purification of heavy metals a formidable challenge.

#### 5.1.2. Ammonia Leaching Method

To overcome the inherent limitations of acid leaching in terms of metal selectivity, researchers have developed the ammonia leaching method for treating ES. This method predominantly employs ammonia or a combination of ammonia and ammonium salt as the leaching agent. The target metal forms soluble ammonia coordination ions within the solution, facilitating its dissolution, while impurity elements either fail to form or exhibit minimal affinity for ammonia coordination ions, thus remaining within the slag. Consequently, this technique enables the desired metal to be effectively separated from its impurities. Notably, the ammonia leaching method exhibited remarkable selectivity for dissolving Cu, Zn, Ni, Co, and Ag while effectively inhibiting the dissolution of Cr and Fe. The process of leaching ESs through the ammonia leaching method encompasses the realm of metal electrochemical corrosion.

R. Salhi [66] utilized the ammonia leaching method to recover nickel and copper from ES. In the low alkaline region, the solubility of nickel and copper, including their hydroxides and four types of ES, is enhanced in the presence of ammonia. The solubility changes with pH at different ammonia concentrations. The optimal ammonia concentration for leaching [NH_3_]_total_ at pH 8 = 11 was found to be less than 6 mol/L. Shi et al. [57] selectively recovered nickel from sludge using the NH_3_-(NH_4_)_2_CO_3_ ammonia leaching system and analyzed the leaching behavior of nickel, iron, and chromium during the ammonia leaching process using Eh-pH diagrams. It was found that nickel can be leached out in the form of the complex [Ni(NH_3_)_n_]^2+^, while iron and chromium are left in the leach residue as precipitates. The optimal leaching conditions were determined to be 28.28 min, 54.07 °C, a liquid-to-solid ratio of 23.7:1, and an NH_3_·H_2_O concentration of 5.10 mol/L. Silva et al. [54] conducted experiments employing both acid leaching and ammonia leaching to treat ES. The results indicated that the leaching efficiency of sulfuric acid on elements such as nickel, zinc, and copper in sludge was significantly greater than that of ammonia or ammonium. However, the concentration of trace elements in the solution increased with increasing sulfuric acid concentration, which posed challenges for the subsequent recovery of valuable metals in the solution.

Ammonia leaching results in good selectivity and is suitable for accessing metal minerals with high alkaline gangue contents. This selectivity is advantageous for subsequent processes of metal separation and purification. Additionally, the ammonia leaching process for copper and nickel recovery from waste ES is relatively brief. However, the complexity of the ammonia leaching process and the high requirements for equipment sealing restrict the practical application of ammonia leaching in production. As shown in Table 4, the difference between acid leaching and ammonia leaching is their effect on the inhibition of iron and chromium in the leaching residue. The inhibition effect of ammonia leaching on iron and chromium is better than that of acid leaching.

### 5.2. Pyrometallurgical Treatment

**Figure 6:**
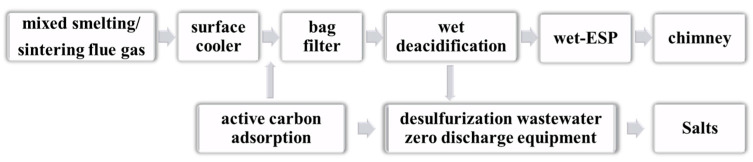
ES pyrometallurgy process.

The pyrometallurgical process is used to treat ES by adding reducing agents and fluxes during the smelting process of the sludge. High-temperature reactions can be used to obtain metals or intermediate products. Roasting–leaching and smelting are the two main pyrometallurgical processes used for treating sludge. Figure 6 illustrates the general process of incinerating ESs.

#### 5.2.1. Roasting and Leaching Methods

During the roasting–leaching process, ES is initially subjected to roasting to remove water, organic matter, and some impurities, reducing the volume of sludge and enriching valuable metals. Then, acid, alkali, water, and other media are used as leaching agents to extract valuable metals from the roasted products. The purpose of roasting pretreatment is determined by the composition of the sludge and the leaching process.

Amaral et al. [60] utilized sulfate roasting of ES and employed sodium thiosulfate as a leaching agent to recover gold, silver, copper, and zinc from the sludge, investigating the metal recovery rates under various experimental conditions. Under experimental conditions, the leaching efficiency of sodium thiosulfate is comparable to that of cyanide. Zhou et al. [41] conducted a study on the roasting–acid leaching method with the addition of sodium sulfate and the supplementation of sodium hydroxide for the recovery of chromium from ES. Under optimal conditions, the recovery rate of chromium can reach 93.35%. The analysis results indicate that the combined effect of sodium sulfate, sodium hydroxide, and air roasting greatly improves the separation and recovery efficiency of chromium. The remaining residue is transformed into a Fenton-like catalyst for the degradation of tetracycline, realizing the efficient utilization of resources. Gustavo Rossini et al. [59] recovered Cu, Zn, and Ni from ES by sulfuric acid acidification roasting. Pyrite waste was used as a vulcanizing agent, mixed with ES for roasting treatment, and then leached with deionized water at room temperature. The recovery rates of Cu, Zn, and Ni are 50%, 60%, and 43%, respectively, which achieves the purpose of treating waste with waste. Yao et al. used an HCl chlorination agent and SiO2 mineralizer to selectively separate and extract Cu from Cu-Ni mixed ES by chlorination and a mineralization surface interface phase transformation method. The isolated Cu is in the form of copper chloride dihydrate, while more than 97% of the nickel is in the form of nickel silicate. The optimum process conditions were 800 °C held for 90 min, the liquid-to-solid ratio was 3:2, the extraction rate of copper was 93%, and the purity of the product was about 91%. The mechanism of copper–nickel hydration separation and extraction is as follows:

NiCl_2_ + H_2_O → NiO + HCl (5)

NiCl_2_ + SiO_2_ + H_2_O → Ni_2_SiO_2_ + HCl_2_ (g) (6)

CuCl_2_ + H_2_O → CuCl_2_ (g) + H_2_O (g) (7)

CuCl_2_ +2H_2_O → Cu(OH)_2_ + 2HCl (g) (8)

Cu(OH)_2_ + HCl(g) → CuCl_2_ + H_2_O (9)

The roasting–leaching process is advantageous for separating and extracting metals that are challenging to separate using the hydrometallurgical process. In comparison to the ammonia leaching method and the acid leaching method, the roasting–leaching process requires a smaller quantity of leaching agents and exhibits superior selectivity toward heavy metals. Nevertheless, the process is hindered by drawbacks such as a lengthy process flow, high energy consumption, substantial production investment, and a low recovery rate of heavy metals, making it challenging to promote and implement heavy metals in practical production.

#### 5.2.2. Smelting Method

The smelting method is a pyrometallurgical treatment process that utilizes coal, coke, and reducing agents as fuel and incorporates additives such as dolomite, limestone, and copper ore to recover metals such as Cu, Zn, and Ni from ES. The selection and quantity of additives, as well as the roasting conditions, significantly impact the smelting process. Tian et al. [67] introduced a refining process using carbon thermal reduction roasting and low carbon oxide reduction to recover valuable metals such as copper, zinc, nickel, tin, and lead from ES. At 1200 °C, 20% carbon (wt%) was added, and after a reaction time of 1 h, 90.77% of the lead, 99.92% of the zinc, and 95.14% of the tin volatilized into the flue gas, resulting in the formation of black copper after roasting. Moreover, at 1300 °C, with the addition of 8% carbon and 6% SiO_2_ (slagging agent) and a reaction time of 3 h, high-purity anode copper with a purity of 98% and water-quenched slag suitable for construction materials can be obtained.

### 5.3. Biological Treatment

**Figure 7:**
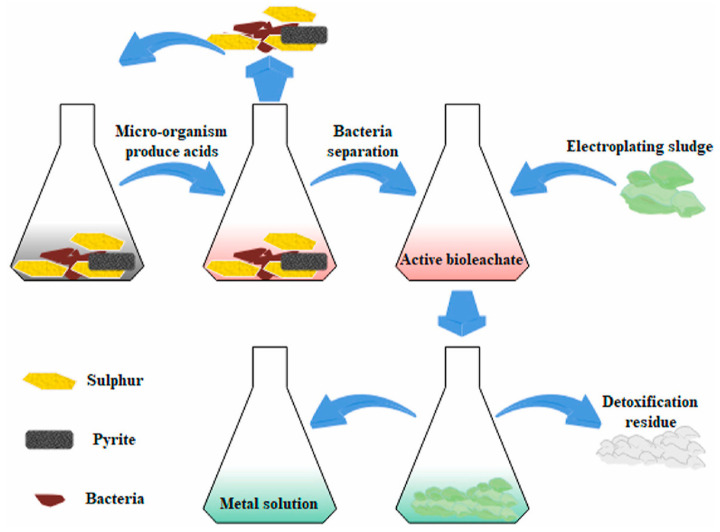
The basic process of bacterial leaching [68] (Elsevier copyright permission).

The biological treatment of ES involves the utilization of specific microorganisms or their byproducts to break down degradable components within solid waste, thereby achieving the objectives of waste recycling and harm reduction. Currently, the prevailing biological methods for treating ES include bioleaching, biosorption, and sludge composting. The biological sludge treatment process is shown in Figure 7.

#### 5.3.1. Biological Leaching

Biological treatment of ES involves the use of specific microorganisms or their byproducts to degrade components in solid waste, thereby achieving the objectives of waste recycling and harm reduction. Currently, the predominant biological methods for treating ES include bioleaching, biosorption, and sludge composting. Among the most common microorganisms are *Acidithiobacillus ferrooxidans* and *Acidithiobacillus thiooxidans*. The metabolic reactions are as follows:

(10) $${4\mathrm{Fe}}^{2+}{+4H}^{+}{+O}_{2}\to {2H}_{2}{O+4\mathrm{Fe}}^{3+}$$

(11) $${\mathrm{MS}+2\mathrm{Fe}}^{3+}\to M^{2+}{+2F}^{2+}+S$$

(12) $${S+O}_{2}{+2H}_{2}O\to {4H}^{+}{+\mathrm{SO}}_{4}^{2-}$$

Liu et al. [69] investigated the regulatory effects of *Acidithiobacillus thiooxidans* and *Acidithiobacillus ferrooxidans* sludge on sludge dewatering. Compared with other methods, such as hydrothermal, ultrasonic, and microwave treatments, the use of ferric chloride and calcium oxide for bioleaching or chemical treatment can significantly improve the dewaterability of urban sewage sludge without reducing the organic components, which is beneficial for reducing the cost of reuse. Yang et al. [38] used *Acidithiobacillus thiooxidans* (A.t.) as a bioleaching agent, with elemental sulfur (S) serving as the energy source for the bacteria. In the A.t-S system, H_2_SO_4_ is generated, which dissolves and releases the target metal. Under optimal conditions, the nickel in ES can be completely leached out. Sathyavathi et al. [62] isolated a strain of nickel-tolerant bacteria (*Microbacterium* sp.) from electroplating wastewater that can convert soluble NiSO_4_ into insoluble NiO nanoparticles. The research results demonstrated the feasibility of the use of the metal-tolerant bacterium *Microbacterium* sp. for converting toxic nickel into less toxic nickel oxide nanoparticles. Compared to traditional methods for heavy metal detoxification, this technique has an exceptionally low cost.

#### 5.3.2. Biological Adsorption

The biosorption method involves the cultivation of microorganisms, such as bacteria and molds, with specific functions under controlled conditions to adsorb valuable metals present in sludge. Due to the diversity of microorganisms, variations exist in the structure of cells and tissues, resulting in differences in the adsorption capacity of metal ions with varying concentrations and properties [70]. Microbial adsorption can be categorized into two main types: direct cell adsorption and metabolite fixation. Cell adsorption occurs when metal ions complex with the surface groups of microbial cell walls, leading to the removal and enrichment of heavy metals in sludge. Metabolite fixation refers to the reaction between metabolites produced during microbial growth and metal ions, causing the adsorption of metal ions.

Selecting appropriate adsorbents is crucial for biosorption. Currently, there are a wide variety of adsorbents available on the market that possess suitable pricing, strong adsorption capacity, ease of heavy metal adsorption and recovery, and environmental friendliness [71]. This method has been successfully employed for the treatment of heavy metal ion-contaminated wastewater.

#### 5.3.3. Sludge Composting

Sludge composting is a process whereby the organic matter in sludge undergoes a series of biochemical reactions under the influence of microorganisms, resulting in its transformation and stabilization. This process results in the production of fertilizer that is beneficial for plant absorption and growth. Research has demonstrated that heavy metals present in sludge can enhance the growth and development of plants, while microorganisms facilitate the absorption of heavy metals by plants. The factors that influence sludge composting include moisture content, temperature, pH, and other variables [72,73]. Additionally, the auxiliary materials employed primarily consist of expansion agents and heavy metal passivation agents. Sludge composting technology offers several advantages, including reduced capital investment, simplified process flow, minimal waste gas and wastewater discharge, and extensive application prospects. Nonetheless, ES exhibits complex compositions and properties and contains heavy metals that are resistant to degradation. Therefore, further development and research are needed to determine whether the utilization of treated sludge as fertilizer will result in secondary pollution [74]. Consequently, stringent quality control measures must be implemented when utilizing ESs as fertilizer.

Microbial treatment technology offers a cleaner and more environmentally advantageous approach than traditional sludge treatment methods. It holds great potential for use in the secondary recovery of low-grade mineral resources. However, the growth of microorganisms in microbial treatment technology is time-consuming due to the toxicity of ES and the shear force exerted by the particles, leading to long treatment cycles and unstable effects [75]. Further research is required to investigate the biological detoxification mechanism, rapid biological treatment, leaching kinetics, and scale of ESs.

### 5.4. Materialization Treatment

The composition of ES is complex, and if a wet process is adopted, subsequent complex metal separation and purification work will be needed. ES can be used as a raw material or auxiliary material for the production and preparation of functional materials, such as ferrites, capacitors, catalysts, adsorbents, and electrode materials.

Electroplating sludge can be utilized in the production of construction materials, and the presence of heavy metal elements in the sludge affects the hydration properties of the solidified materials. Xiao et al. [76] employed an enhanced deionization ion (EDI) structure for continuous treatment of simulated electroplating wastewater containing nickel. The removal efficiency for Ni^2+^, Cu^2+^, Zn^2+^, Cd^2+^, and Cr^3+^ in electroplating rinse water reached a minimum of 99.8%, allowing for recycling within the electroplating industry and reducing the discharge of electroplating sludge at its source. This led to improved quality of the sludge and reduced costs associated with its treatment and transportation, as well as significant economic benefits.

Ferrite is a high-performance magnetic material that can be divided into two categories: simple ferrite and complex ferrite. It possesses stable chemical properties and is not easily soluble in water, acid, alkali, or salt solutions. During the treatment of ES, heavy metal ions (such as Mn^2+^, Cu^2+^, Ni^2+^, Zn^2+^, and Co^2+^) can react with Fe^3+^ and Fe^2+^ to form stable ferrites, which can be firmly fixed in the spinel ferrite lattice [77]. Chen et al. [78] used a hydrothermal method to treat ES containing copper, zinc, iron, chromium, and nickel. FeCl·6H_2_O was added as the iron source, and NaOH was used as the precipitant. Using this method, they successfully prepared copper-rich Ni-Zn-Cr ferrite and recovered copper through an extraction method, with a recovery rate ranging from 76% to 84%. Weng et al. [30] used nickel-rich ES as a raw material and added an iron source and modifier (Na_2_CO_3_) to prepare nickel ferrite through a hydrothermal acid washing method. After drying, they obtained a high-performance lithium battery negative electrode material, NiFe_2_O_4_.

Nickel, as one of the vital metals in modern industry, exhibits significant application potential in supercapacitors through its oxides, hydroxides, and nickel-based layered double hydroxide (LDH) materials [79,80,81]. Hou et al. [82] utilized nickel-containing ES as the raw material and urea as the precipitant to synthesize Al and Fe codoped α-Ni(OH)_2_ and Ni(HCO_3_)_2_ through a hydrothermal method. The nickel-based composite nanomaterial exhibited excellent electrochemical performance, and the supercapacitor prepared from it had a specific capacitance of 495.6 C·g^−1^, maintaining 55.58% of its initial capacitance after 3500 cycles. Liu et al. [83] utilized hydrochloric acid to leach Ni^2+^, Fe^3+^, and Al^3+^ from nickel-containing ES. Urea was used as a precipitant to synthesize two-dimensional layered nickel-based LDH through a hydrothermal method. The LDH exhibited a specific capacitance of 1652.20 F·g^−1^ at a current density of 0.5 A·g^−1^ and maintained a capacitance of 766.69 F·g^−1^ after 1000 cycles. The study confirmed that the content of -O-C≡N in LDH increased with the addition of urea, and excessive urea led to the transformation of LDH into Ni(HCO_3_)_2_.

ES typically contains heavy metals, flocculants, precipitates, etc. These materials can be prepared into adsorbents through activation and calcination treatment, the adsorption mechanism of which involves mainly ion exchange and surface coordination. Eun-Ji Cho et al. [84] utilized the Fenton oxidation process to treat ES. The obtained Fenton sludge was activated at various temperatures to produce active adsorbents for the removal of Cd^2+^ from wastewater. When TA–FS–900 was applied to wastewater discharged from a zinc smelter, 98.4% of the Cd was removed from the wastewater at a dosage of 10 g/L. Liu et al. [40] utilized ES containing Co and Cr, and by adding Na, rod-shaped erbium crystals were synthesized at 160 °C for 10 h in a high-pressure reactor. Long rod-shaped erbium crystals with a concentration of 0.3 g/L can remove more than 99% of the Zn and Cu in electroplating wastewater, 37.9% of the Cu, and 53.3% of the Ni, exhibiting a greater removal efficiency than powdered activated carbon, poly aluminum chloride, polyferric sulfate, or pure Na_2_S·9H_2_O reagent. The treated electroplating wastewater met the discharge standard.

The utilization of ES for the preparation of construction materials such as cement, sintered bricks, and ceramics has been extensively studied. The main focus of the related research is to improve the performance of construction materials through the use of additives and evaluate the risk of metal leaching. ES contains various heavy metals, which can form new crystal phases during cement production. However, due to the complexity of heavy metals, interactions between them and their crystal phases still require further investigation. During the sintering process, most of the ES is immobilized in the cement. Heavy metal ions can affect the performance of cement, and the heavy metal ions in ES directly influence the production and usage performance of cement; however, the problem of excessive heavy metal content also exists. Many researchers have conducted toxicity leaching tests on ES as a partial substitute for cement clinker, and the results indicate that it poses no significant threat to the environment [44,48,85].

ESs include numerous metal oxides, sulfates, phosphates, hydroxides, and silicates; thus, these materials serve as excellent catalyst precursors and can be utilized as raw materials for catalyst fabrication. The ES often exhibits vibrant colors, which are attributed to the presence of multiple colored metal ions in the sludge. The color of ES is related to the valence states of the ions, such as Cu^2+^, Cr^3+^, and Ni^2+^, which typically result in a green color. It can be dried and finely ground to serve as an alternative green pigment in decorative mortar production.

In summary, the materialization technology used for ES not only enables the full utilization of heavy metals in sludge to produce high-value-added products but also provides a solution for the large-scale disposal of sludge, making this process a hot topic in the research of ES treatment and disposal techniques. The key challenge of this technology lies iSn the variable composition of the sludge, which requires preliminary analysis and rational utilization based on its specific characteristics, which involves a high initial investment. However, the further development and application of materialization of ES are constrained by issues such as secondary pollution during the treatment process, long-term stability of the materials, imperfect policies, and insufficient market confidence.

### 5.5. Technical Comparison

In summary, there are various methods available for the treatment and disposal of ES, including hydrometallurgical processes, pyrometallurgical processes, biological treatment, and solidification. However, each treatment method has limitations and cannot make full use of ES. The advantages and disadvantages of various ES treatment processes are shown in Table 5.

The hydrometallurgical process offers a high recovery rate and requires minimal equipment investment. However, the wet extraction process involves a significant quantity of costly chemical reagents and generates substantial wastewater during operation. During ammonia leaching, inadequate equipment sealing may result in the production of waste gas, necessitating strict equipment sealing measures. Pyrometallurgical treatment offers high production capacity and comprehensive treatment effects but requires substantial capital investment. The operation of the equipment generates a significant amount of flue gas. The pyrometallurgical process is strongly influenced by the type and quantity of additives. In addition, ES is characterized by high water content, low calorific value, low metal content, and complex composition, and these conditions pose challenges, including high energy consumption, difficult flue gas treatment, low metal recovery rate, and limited variety of recovered metals. Biological treatment of ESs offers significant advantages in mitigating environmental pollution. It exhibits a strong metal adsorption capacity, high recovery rate for heavy metals, low capital investment, simple operation process, absence of secondary pollution, and promising application prospects. However, this method is hindered by the lengthy process of selecting and cultivating suitable strains, as well as the slow treatment speed, thereby constraining the industrial application of biological treatment technology. The materialized treatment of ES offers advantages such as simple equipment, low cost, reduced volume, land resource utilization, high-value-added product production, and significant economic benefits. This topic represents a focal point in the research on ES disposal technology. However, the long-term stability of ES treatment through materialization technology requires further investigation. It is crucial to improve research on the curative effects of materials to ensure product quality.

## 6. Conclusions and Perspectives

ESs have both harmful and resource value. Their large emissions and accumulation have caused significant damage to the environment, seriously hindering the healthy development of the electroplating industry. Extensive and in-depth research has been conducted by scholars on the harm and resource of ES. They have developed treatment methods suitable for various forms of electroplating sludge, including sludge solidification technology, pyrometallurgy, hydrometallurgy, high-value utilization, and other technical means. These treatment processes offer significant economic value and environmental benefits and can promote the sustainable development of electroplating enterprises. The research on resource utilization of ES in China is still in its early stages, with great potential for reducing sludge treatment costs, preventing secondary pollution, and achieving comprehensive sludge utilization.

The treatment and disposal of ESs has become a research focus for scientists, leading to the development of various treatment methods. These include solidification techniques, thermal treatment methods, recovery of valuable elements, and material utilization. However, further improvement is still required to meet the demands of efficient and sustainable ES management. To enhance the solidification effect of ESs, it is necessary to develop more efficient additives and effectively control heavy metal loss during the solidification/stabilization treatment. In addition, the thermal treatment process should explore more efficient and energy-saving conditions to reduce energy consumption while improving treatment effectiveness. Furthermore, wet metallurgical treatment should minimize the use of chemical reagents and decrease wastewater generation. Materialization technology aims to achieve more stable treatment effects by fully utilizing the resources of ESs. Lastly, the biological method involves developing functional bacteria to improve efficiency in ES treatment. ESs should be treated and disposed of according to the specific characteristics and process conditions of the sludge. To achieve comprehensive utilization of different components within the sludge, it is essential to transition from a single technology to a multi-process approach. Additionally, electroplating wastewater can be treated using an EDI structure. This reduces the discharge of electroplating sludge from the source and makes electroplating sludge treatment less difficult. There is an inadequate understanding of the nature of ES, necessitating further research focusing on fundamental theories encompassing physicochemical properties, phase characteristics, and forms of heavy metal occurrence in ESs. Furthermore, it is crucial to enhance regulation and utilization practices for ES by enacting relevant laws, regulations, and industry standards that govern the management and utilization of ES from a legal perspective. To implement these regulations, multiple departments must collaborate while clarifying individual responsibilities.

## Figures and Tables

**Table 1:** Element concentrations (% dry mass) and metal prices in ES.

Metal	Minimum	Maximum	Metal Price per Ton ($) *
Cu	0.0003	60.2	8736
Zn	0.0003	55.7	3529
Ni	<0.0005	36.0	26,560
Sn	0.0007	3.76	25,207
Pb	<0.0002	16.2	2106
Al	0.00007	35.4	2569
Cr	<0.001	28.5	9530–10,221

* Changjiang Nonferrous Metal network.

**Table 2:** Common types and components of ES.

Types of Sludge	Plating	w/%							Processing Method
		Ca	Cr	Fe	Ni	Cu	Zn	Sn	
Separate sludge	Ni	0.69		1.55	96.2				Nickel-based composite nanomaterials [22]
	Cr	13.8	9.77	1.42			0.33		Chromium oxide nanocomposites [23]
			62.9	11.8	3.58	2.6	6.5		Pigment synthesis [24]
		9.0	6.3	12.0	4.00	3.0	6.0		Metal–organic framework [25]
			3.94	7.52	6.13	5.14			Crude stainless [26]
	Cu	16.1		16.1		49.9	0.11	2.91	Catalyst [27]
		5.7	0.1	1.5	0.3	3.8	1.2		Smelting [28]
	Sn			24.4				4.0	Sn@C Nanorodzn
	Zn			12.6			8.7		Flocculant [20]
Mixed sludge	Cr-Ni-Zn	3.74	2.94	13.6	4.94	1.18	7.49		Sorbent [29]
	Cu-Cr	12.44	3.91	7.55	6.02	5.13	12.44		Solidify [6]
	Cu-Cr-Ni	13.3	2.06	5.57	6.09	3.09			Ferrite [30]
			4.22		7.27	6.51			Oxidation roasting, water immersion [31]
		15.38	0.09	12.87	0.41	12.94	0.04		Bioleaching [32]
	Cu-Ni	18.62		3.01	5.51	6.93	1.6		Chlorination–mineralization–volatilization [33]

**Table 3:** Various methods used to extract metals from ESs.

Treatment Approaches	Reagents and Operations	Efficiency
Acid leaching	H_2_SO_4_, added NaOH until the pH of 7 and 14	98.99% Cu, 99.27% Ni and 97.22% Cr [52]
	HNO_3_, HCl, hydrothermally	99.8% Fe, 96.6% Al, and 98.7% Zn [20]
	HNO_3_ 0-0.65 ppm, sonication 0–20 min,	82.2% Zn, 87.3% Pb and 9.5% Cu [53]
	H_2_SO_4_ 100 g/L, liquid-to-solid ratio 5:1, particle size <1 mm, digestion time 1 h	88.6% Cu, 98.0% Ni and 99.2% Zn [54]
	H_2_O_2_, H_2_SO_4_, ultrasonic, two-stage acid leaching	97.42% Cu, 98.46% Ni, 98.63% Zn, 98.32% Cr and 100% Fe [55]
	EDTA 70 mM, citric acid 10 mM, electrodeposition	82.21% Cu [56]
Ammonia leaching	NH_3_-(NH_4_)_2_CO_3_, liquid-to-solid ratio 23.7:1, NH_3_·H_2_O 5.10 mol/L, 28.28 min	98.04% Ni [57]
	NH_3_ 20%, pH 10.8–11.2, 90 min	70% Cu, 50% Zn and Ni [58]
Roasting and leaching	chlorination roasting, (NH_4_)_2_CO_3_, H_2_O_2_, 60 °C	99% Zn, 98% Cu and 96% Cr [59]
	sulfating roasting, galvanic sludge/pyrite ratio 1:0.4, roasting 90 min, 550 °C	60% Zn, 43% Ni and 50% Cu [54]
	sulfate roasting, sulfate roasting, sludge/sulfur 1:0.4, 550 °C 90 min, water leaching 15 min	80% Ag, 63% Cu and 73% Zn [60]
Bioleaching	*Sulfobacillus acidophilus*, *Acidithiobacillus caldus*	94.7%Cd, 99%Cr, 98.8%Cu, 97.4%Mn, 98.9%Ni and 78.7%Zn [32]
	*Acidthiobacillus thiooxidans*, *Acidithiobacillus ferrooxidans*, pH = 1.0, 30 °C, S = 0.8 g/L	100%Ni [61]
	Nickel-resistant bacteria, 0.8% NaCl, 30 °C	95%Ni [62]

**Table 4:** Comparison of the main features of acid leaching and ammonia leaching.

Characteristic	Acid Leaching	Ammonia Leaching
Inhibition of Fe and Cr	Inefficient	Effective
Selectivity	Low	High
Leaching Efficiency	Varies depending on the metal being leached	Generally high
Environmental Impact	Acid pollution	Ammonia leak
Equipment	Corrosive to equipment	Requires well-sealed equipment
Health and Safety Risks	Acid fumes, wastewater	Ammonia gas, wastewater
Recycling of Leaching Agents	Challenging	Feasible

**Table 5:** Comparison of ES resource disposal technologies.

	Pyrometallurgical	Hydrometallurgical	Pyrometallurgical + Hydrometallurgical	Materialization	Biological
Equipment investment	large	lesser	large	lesser	small
Production capacity	large	small	lesser	large	small
Environmental protection	flue gas, pollution, less wastewater	waste gas, acid, and alkali wastewater	flue gas and wastewater	exhaust gas	no waste
Running cost	high	high	high	high	low
Types of metals recovered	single	various	various	none	none
Floor space	large	large	large	lesser	small

## Data Availability

The data in this study are presented in the article.

## References

[1] Magalhães J.M., Silva J.E., Castro F.P., Labrincha J.A. (2005). Physical and chemical characterisation of metal finishing industrial wastes. J. Environ. Manag..

[2] Yu Y., Huang Q., Zhou J., Wu Z., Deng H., Liu X., Lin Z. (2021). One-step extraction of high-purity CuCl2·2H2O from copper-containing electroplating sludge based on the directional phase conversion. J. Hazard. Mater..

[3] Wang H., Liu X., Zhang Z. (2024). Approaches for electroplating sludge treatment and disposal technology: Reduction, pretreatment and reuse. J. Environ. Manag..

[4] Qu Z., Su T., Zhu S., Chen Y., Yu Y., Xie X., Yang J., Huo M., Bian D. (2021). Stepwise extraction of Fe, Al, Ca, and Zn: A green route to recycle raw electroplating sludge. J. Environ. Manag..

[5] Qu Z., Chen Y., Chen Y., Zhu S., Liu J., Ren H., Su T., Huo M. (2023). Efficient separation of impurities Fe/Al/Ca and recovery of Zn from electroplating sludge using glucose as reductant. Sci. Total Environ..

[6] Yong Y., Hua W., Jianhang H. (2021). Co-treatment of electroplating sludge, copper slag, and spent cathode carbon for recovering and solidifying heavy metals. J. Hazard. Mater..

[7] Datsenko V., Borzenko O., Kaliuzhna I. (2021). Vertical Migration of Copper and Zinc Ions in Soils Polluted by Electroplating Sludge. CLEAN Soil Air Water.

[8] Yuan H., Deng L., Cai X., Zheng T., Zhou S., Chen Y., Yuan Y. (2016). Recycling electroplating sludge to produce sustainable electrocatalysts for the efficient conversion of carbon dioxide in a microbial electrolysis cell. Electrochim. Acta.

[9] Yu J.-x., Li H.-x., Zhou R.-y., Li X.-d., Wu H.-j., Xiao C.-q., Chi R.-A. (2022). Surface ion imprinted bagasse for selective removal of Cu (II) from the leaching solution of electroplating sludge. Colloids Surf. A Physicochem. Eng. Asp..

[10] Singh A., Sharma R.K., Agrawal M., Marshall F.M. (2010). Health risk assessment of heavy metals via dietary intake of foodstuffs from the wastewater irrigated site of a dry tropical area of India. Food Chem. Toxicol..

[11] Huyen P.T., Dang T.D., Tung M.T., Huyen N.T.T., Green T.A., Roy S. (2016). Electrochemical copper recovery from galvanic sludge. Hydrometallurgy.

[12] Lee J.-C., Pandey B.D. (2012). Bio-processing of solid wastes and secondary resources for metal extraction—A review. Waste Manag..

[13] Chen D.T., Au W.Y., van Ewijk S., Roy A., Stegemann J.A. (2021). Elemental and mineralogical composition of metal-bearing neutralisation sludges, and zinc speciation—A review. J. Hazard. Mater..

[14] Krishnan S., Zulkapli N.S., Kamyab H., Taib S.M., Din M.F.B.M., Majid Z.A., Chaiprapat S., Kenzo I., Ichikawa Y., Nasrullah M. (2021). Current technologies for recovery of metals from industrial wastes: An overview. Environ. Technol. Innov..

[15] Su J., Jin G., Li C., Zhu X., Dou Y., Li Y., Wang X., Wang K., Gu Q. (2014). Ultrasonic preparation of nano-nickel/activated carbon composite using spent electroless nickel plating bath and application in degradation of 2,6-dichlorophenol. J. Environ. Sci..

[16] Vilarinho I.S., Carneiro J., Pinto C., Labrincha J.A., Seabra M.P. (2021). Development of Coloured Stoneware Bodies through the Incorporation of Industrial Cr/Ni Electroplating Sludge. Sustainability.

[17] Tian Q., Dong B., Guo X., Xu Z., Wang Q., Li D., Yu D. (2021). Comparative atmospheric leaching characteristics of scandium in two different types of laterite nickel ore from Indonesia. Miner. Eng..

[18] Huang Q., Wang Q., Liu X., Li X., Zheng J., Gao H., Li L., Xu W., Wang S., Xie M. (2022). Effective separation and recovery of Zn, Cu, and Cr from electroplating sludge based on differential phase transformation induced by chlorinating roasting. Sci. Total Environ..

[19] Tavares C.R.G., de Melo Franco J. (2012). Production of concrete paving blocks (CPB) utilising electroplating residues—Evaluation of mechanical and micro-structural properties. Can. J. Chem. Eng..

[20] Bian R., Su T., Gao Y., Chen Y., Zhu S., Liu C., Wang X., Qu Z., Zhang Y., Zhang H. (2022). Enrichment and recycling of Zn from electroplating wastewater as zinc phosphate via coupled coagulation and hydrothermal route. Arab. J. Chem..

[21] Benvenuti T., Krapf R.S., Rodrigues M.A.S., Bernardes A.M., Zoppas-Ferreira J. (2014). Recovery of nickel and water from nickel electroplating wastewater by electrodialysis. Sep. Purif. Technol..

[22] Hou Z., Meng H., Shao X., Wang X., Tahir M.U., Ahmad S., Yang C., Su X., Zhang L. (2022). Synthesis of amorphous hollow Ni(HCO3)2 nanostructures with excellent supercapacitor performance from nickel-containing electroplating sludge. J. Environ. Chem. Eng..

[23] Li Y., Zhang H., Shao L.-M., He P.-J. (2015). Preparation of a metal-phosphate/chromium oxide nanocomposite from Cr(III)-containing electroplating sludge and its optical properties as a nanopigment. Process Saf. Environ. Prot..

[24] Matović L., Vujasin R., Kumrić K., Krstić S., Wu Y.-N., Kabtamu D.M., Devečerski A. (2021). Designing of technological scheme for conversion of Cr-rich electroplating sludge into the black ceramic pigments of consistent composition, following the principles of circular economy. J. Environ. Chem. Eng..

[25] Kabtamu D.M., Wu Y.-N., Chen Q., Zheng L., Otake K.-I., Matović L., Li F. (2020). Facile Upcycling of Hazardous Cr-Containing Electroplating Sludge into Value-Added Metal–Organic Frameworks for Efficient Adsorptive Desulfurization. ACS Sustain. Chem. Eng..

[26] Heng W., Yong Y., Jianhang H., Hua W. (2024). A novel method for effective solidifying chromium and preparing crude stainless steel from multi-metallic electroplating sludge. J. Hazard. Mater..

[27] Zhou Z., Liu T., Wu J., Li H., Chu S., Zhu X., Zhang L., Lu J., Ivanets A., Davronbek B. (2023). Preparation of copper-based catalysts from electroplating sludge by ultrasound treatment and their antibiotic degradation performance. Environ. Res..

[28] Jandova J., Štefanová T.Á., Niemczyková R. (2000). Recovery of Cu-concentrates from waste galvanic copper sludges. Hydrometallurgy.

[29] Stefanova R.Y. (2000). Sorption of metal ions from aqueous solutions by thermally activated electroplating sludge. J. Environ. Sci. Health Part A.

[30] Weng C., Sun X., Han B., Ye X., Zhong Z., Li W., Liu W., Deng H., Lin Z. (2020). Targeted conversion of Ni in electroplating sludge to nickel ferrite nanomaterial with stable lithium storage performance. J. Hazard. Mater..

[31] Xiao Y., Li L., Huang M., Liu Y., Xu J., Xu Z., Lei Y. (2022). Treating waste with waste: Metals recovery from electroplating sludge using spent cathode carbon combustion dust and copper refining slag. Sci. Total Environ..

[32] Sun J., Zhou W., Zhang L., Cheng H., Wang Y., Tang R., Zhou H. (2021). Bioleaching of Copper-Containing Electroplating Sludge. J. Environ. Manag..

[33] Yu Y., Ge J., Wu Z., Lin J., Zhu Z., Yang Q., Liu X. (2023). One-step extraction of CuCl2 from Cu-Ni mixed electroplating sludge by chlorination-mineralization surface-interface phase change modulation. Surf. Interfaces.

[34] Wu J.-Y., Chou W.-S., Chen W.-S., Chang F.-C., Shen Y.-H., Chang J.-E., Tsai M.-S. (2012). Recovery of cupric oxide from copper-containing wastewater sludge by acid leaching and ammonia purification process. Desalination Water Treat..

[35] Nair A., Juwarkar A.A., Devotta S. (2008). Study of speciation of metals in an industrial sludge and evaluation of metal chelators for their removal. J. Hazard. Mater..

[36] Shi W., Liu C., Ding D., Lei Z., Yang Y., Feng C., Zhang Z. (2013). Immobilization of heavy metals in sewage sludge by using subcritical water technology. Bioresour. Technol..

[37] Liu W., Li J., Zheng J., Song Y., Shi Z., Lin Z., Chai L. (2020). Different Pathways for Cr(III) Oxidation: Implications for Cr(VI) Reoccurrence in Reduced Chromite Ore Processing Residue. Environ. Sci. Technol..

[38] Dai Z., Wu Y., Hu L., Zhang W., Mao L. (2019). Evaluating physical-mechanical properties and long periods environmental risk of fired clay bricks incorporated with electroplating sludge. Constr. Build. Mater..

[39] Zhang M., Chen C., Mao L., Wu Q. (2018). Use of electroplating sludge in production of fired clay bricks: Characterization and environmental risk evaluation. Constr. Build. Mater..

[40] Liu Y., Khan A., Wang Z., Chen Y., Zhu S., Sun T., Liang D., Yu H. (2020). Upcycling of Electroplating Sludge to Prepare Erdite-Bearing Nanorods for the Adsorption of Heavy Metals from Electroplating Wastewater Effluent. Water.

[41] Zhou Z., Zhang L., Yan B., Wu J., Kong D., Romanovski V., Ivanets A., Li H., Chu S., Su X. (2024). Removal of chromium from electroplating sludge by roasting-acid leaching and catalytic degradation of antibiotics by its residue. J. Environ. Chem. Eng..

[42] Wang X., Ding C., Long H., Wu Y., Jiang F., Chang R., Xue S., Wu J., Cheng K. (2023). A novel approach to treating nickel-containing electroplating sludge by solidification with basic metallurgical solid waste. J. Mater. Res. Technol..

[43] Babel S., del Mundo Dacera D. (2006). Heavy metal removal from contaminated sludge for land application: A review. Waste Manag..

[44] Shih P.-H., Chang J.-E., Lu H.-C., Chiang L.-C. (2005). Reuse of heavy metal-containing sludges in cement production. Cem. Concr. Res..

[45] Chen H., Yuan H., Mao L., Hashmi M.Z., Xu F., Tang X. (2020). Stabilization/solidification of chromium-bearing electroplating sludge with alkali-activated slag binders. Chemosphere.

[46] Orescanin V., Mikulic N., Mikelic I.L., Posedi M., Kampic S., Medunic G. (2009). The bulk composition and leaching properties of electroplating sludge prior/following the solidification/stabilization by calcium oxide. J. Environ. Sci. Health Part A.

[47] Qian G., Yang X., Dong S., Zhou J., Sun Y., Xu Y., Liu Q. (2009). Stabilization of chromium-bearing electroplating sludge with MSWI fly ash-based Friedel matrices. J. Hazard. Mater..

[48] Asavapisit S., Chotklang D. (2004). Solidification of electroplating sludge using alkali-activated pulverized fuel ash as cementitious binder. Cem. Concr. Res..

[49] Mao L., Gao B., Deng N., Zhai J., Zhao Y., Li Q., Cui H. (2015). The role of temperature on Cr(VI) formation and reduction during heating of chromium-containing sludge in the presence of CaO. Chemosphere.

[50] Ramachandran K., Kikukawa N. (2000). Plasma in-flight treatment of electroplating sludge. Vacuum.

[51] Wang J., Zhang S., Qian C., Cui Y., Shi G., Cheng J., Li X., Xin B. (2023). Heat treatment-enhanced bioleaching of new electroplating sludge containing high concentration of CuS and its mechanisms. Sep. Purif. Technol..

[52] Sim H.H., Rafatullah M., Alosaimi A.M., Hussein M.A. (2022). Copper, chromium and nickel recovery from electroplating sludge using acid digestion method. Int. J. Environ. Anal. Chem..

[53] Deng J., Feng X., Qiu X. (2009). Extraction of heavy metal from sewage sludge using ultrasound-assisted nitric acid. Chem. Eng. J..

[54] Silva J., Soares D., Paiva A., Labrincha J., Castro F. (2005). Leaching behaviour of a galvanic sludge in sulphuric acid and ammoniacal media. J. Hazard. Mater..

[55] Li C., Xie F., Ma Y., Cai T., Li H., Huang Z., Yuan G. (2010). Multiple heavy metals extraction and recovery from hazardous electroplating sludge waste via ultrasonically enhanced two-stage acid leaching. J. Hazard. Mater..

[56] Deng D., Deng C., Liu T., Xue D., Gong J., Tan R., Mi X., Gong B., Wang Z., Liu C. (2022). Selective recovery of copper from electroplating sludge by integrated EDTA mixed with citric acid leaching and electrodeposition. Sep. Purif. Technol..

[57] Shi C., Zuo X., Yan B. (2022). Selective recovery of nickel from stainless steel pickling sludge with NH3-(NH4)2CO3 leaching system. Environ. Technol..

[58] Xu W., Liu W., Zhu H., Xu J., Li G., Fu D., Luo L. (2015). Highly Selective Copper and Nickel Separation and Recovery from Electroplating Sludge in Light Industry. Pol. J. Environ. Stud..

[59] Rossini G., Bernardes A. (2006). Galvanic sludge metals recovery by pyrometallurgical and hydrometallurgical treatment. J. Hazard. Mater..

[60] Amaral F.A.D., dos Santos V.S., Bernardes A.M. (2014). Metals recovery from galvanic sludge by sulfate roasting and thiosulfate leaching. Miner. Eng..

[61] Yang Y., Liu X., Wang J., Huang Q., Xin Y., Xin B. (2015). Screening Bioleaching Systems and Operational Conditions for Optimal Ni Recovery from Dry Electroplating Sludge and Exploration of the Leaching Mechanisms Involved. Geomicrobiol. J..

[62] Sathyavathi S., Manjula A., Rajendhran J., Gunasekaran P. (2014). Extracellular synthesis and characterization of nickel oxide nanoparticles from *Microbacterium* sp. MRS-1 towards bioremediation of nickel electroplating industrial effluent. Bioresour. Technol..

[63] Yuxin Z., Ting S., Hongyu C., Ying Z., Zhi G., Suiyi Z., Xinfeng X., Hong Z., Yidi G., Yang H. (2023). Stepwise recycling of Fe, Cu, Zn and Ni from real electroplating sludge via coupled acidic leaching and hydrothermal and extraction routes. Environ. Res..

[64] Li P.P., Peng C.S., Li F.M., Song S.X., Juan A.O. (2011). Copper and Nickel Recovery from Electroplating Sludge by the Process of Acid-leaching and Electro-depositing. Int. J. Environ. Res..

[65] Lee J.-c., Kim E.-y., Kim J.-H., Kim W., Kim B.-S., Pandey B.D. (2011). Recycling of WC–Co hardmetal sludge by a new hydrometallurgical route. Int. J. Refract. Met. Hard Mater..

[66] Salhi R. (2013). Recovery of nickel and copper from metal fInishing hydroxide sludges by ammoniacal leaching. Miner. Process. Extr. Metall..

[67] Tian L., Chen L., Gong A., Wu X., Cao C., Liu D., Chen Z.-Q., Xu Z.-F., Liu Y. (2019). Separation and Extraction of Valuable Metals from Electroplating Sludge by Carbothermal Reduction and Low-Carbon Reduction Refining. Jom.

[68] Tian B., Cui Y., Qin Z., Wen L., Li Z., Chu H., Xin B. (2022). Indirect bioleaching recovery of valuable metals from electroplating sludge and optimization of various parameters using response surface methodology (RSM). J. Environ. Manag..

[69] Liu F., Zhou J., Wang D., Zhou L. (2012). Enhancing sewage sludge dewaterability by bioleaching approach with comparison to other physical and chemical conditioning methods. J. Environ. Sci..

[70] Ye J., Yin H., Mai B., Peng H., Qin H., He B., Zhang N. (2010). Biosorption of chromium from aqueous solution and electroplating wastewater using mixture of Candida lipolytica and dewatered sewage sludge. Bioresour. Technol..

[71] Pathak A., Dastidar M.G., Sreekrishnan T.R. (2009). Bioleaching of heavy metals from sewage sludge: A review. J. Environ. Manag..

[72] Tian X., Qin W., Zhang Y., Liu Y., Lyu Q., Chen G., Feng Z., Ji G., Yan Z. (2024). The inoculation of thermophilic heterotrophic nitrifiers improved the efficiency and reduced ammonia emission during sewage sludge composting. Chem. Eng. J..

[73] Liu Z., Wang X., Li S., Bai Z., Ma L. (2022). Advanced composting technologies promotes environmental benefits and eco-efficiency: A life cycle assessment. Bioresour. Technol..

[74] González I., Robledo-Mahón T., Silva-Castro G.A., Rodríguez-Calvo A., Gutiérrez M.C., Martín M.Á., Chica A.F., Calvo C. (2016). Evolution of the composting process with semi-permeable film technology at industrial scale. J. Clean. Prod..

[75] Kuroda K., Katahira T., Yamada M., Uezono I., Nakamura N., Yamaguchi T., Yamauchi M. (2023). Co-composting of sewage sludge with plant biomass, and analysis of microbiome relevant to plant growth promotion. Bioresour. Technol. Rep..

[76] Feng X., Wu Z., Chen X. (2007). Removal of metal ions from electroplating effluent by EDI process and recycle of purified water. Sep. Purif. Technol..

[77] Chen D., Hou J., Yao L.-h., Jin H.-m., Qian G.-R., Xu Z.P. (2010). Ferrite materials prepared from two industrial wastes: Electroplating sludge and spent pickle liquor. Sep. Purif. Technol..

[78] Chen D., Yu Y.-Z., Zhu H.-J., Liu Z.-Z., Xu Y.-F., Liu Q., Qian G.-R. (2008). Ferrite process of electroplating sludge and enrichment of copper by hydrothermal reaction. Sep. Purif. Technol..

[79] Guan X., Huang M., Yang L., Wang G., Guan X. (2019). Facial design and synthesis of CoSx/Ni-Co LDH nanocages with rhombic dodecahedral structure for high-performance asymmetric supercapacitors. Chem. Eng. J..

[80] Shen S., Liu Y., Zhai D., Qian G. (2020). Electroplating sludge-derived spinel catalysts for NO removal via NH3 selective catalysis reduction. Appl. Surf. Sci..

[81] Zang X., Dai Z., Guo J., Dong Q., Yang J., Huang W., Dong X. (2016). Controllable synthesis of triangular Ni(HCO_3_)_2_ nanosheets for supercapacitor. Nano Res..

[82] Hou Z., Liu T., Usman Tahir M., Ahmad S., Shao X., Yang C., He B., Su X. (2021). Facile conversion of nickel-containing electroplating sludge into nickel-based multilevel nano-material for high-performance pseudocapacitors. Appl. Surf. Sci..

[83] Liu T., Zhou H., Zhong G., Yan X., Su X., Lin Z. (2021). Synthesis of NiFeAl LDHs from electroplating sludge and Their excellent supercapacitor performance. J. Hazard. Mater..

[84] Cho E.-J., Kang J.-K., Lee C.-G., Bae S., Park S.-J. (2023). Use of thermally activated Fenton sludge for Cd removal in zinc smelter wastewater: Mechanism and feasibility of Cd removal. Environ. Pollut..

[85] Ract P.G., Espinosa D.C.R., Tenório J.A.S. (2003). Determination of Cu and Ni incorporation ratios in Portland cement clinker. Waste Manag..

